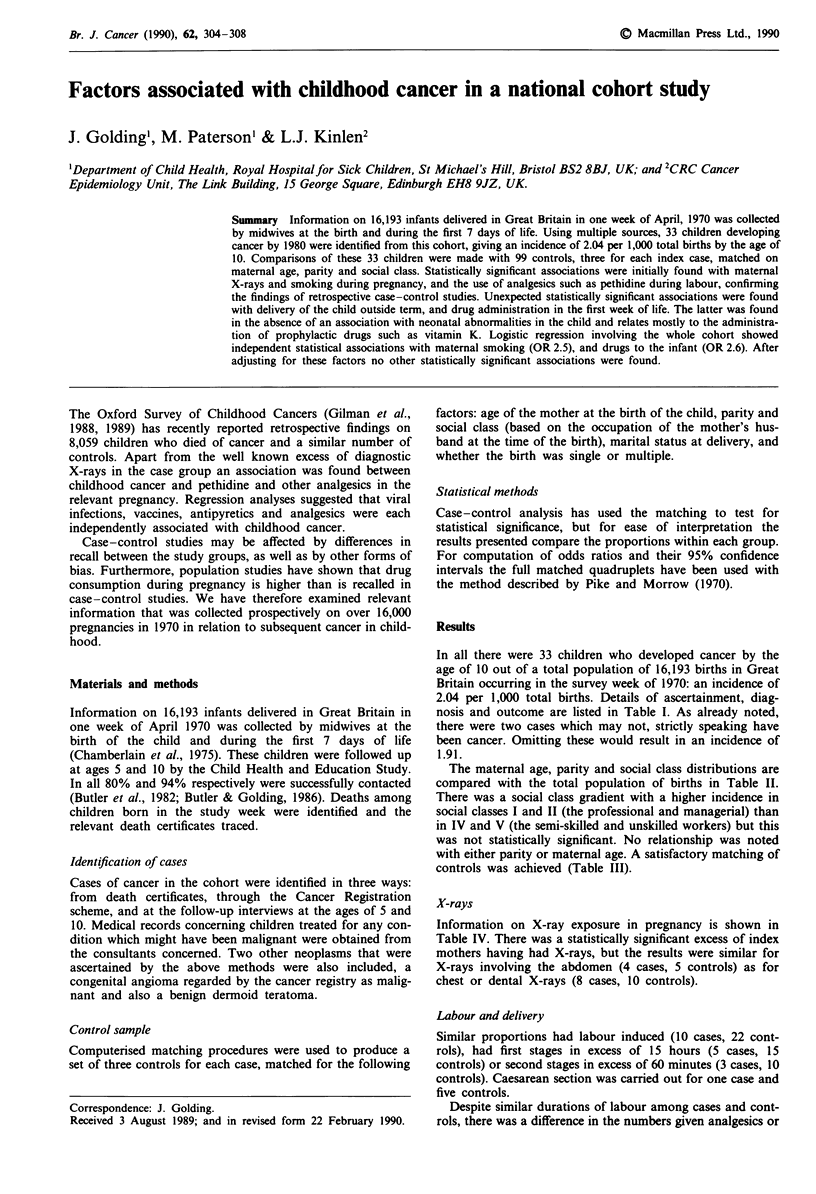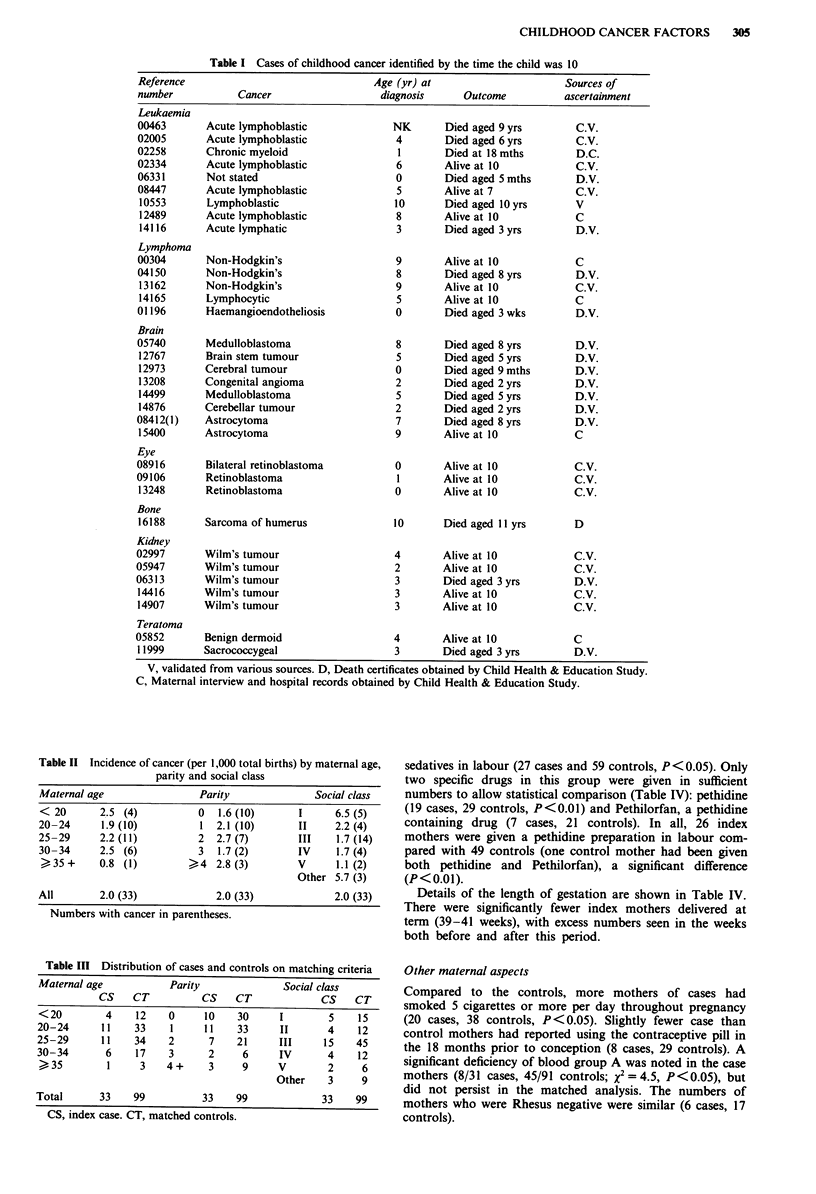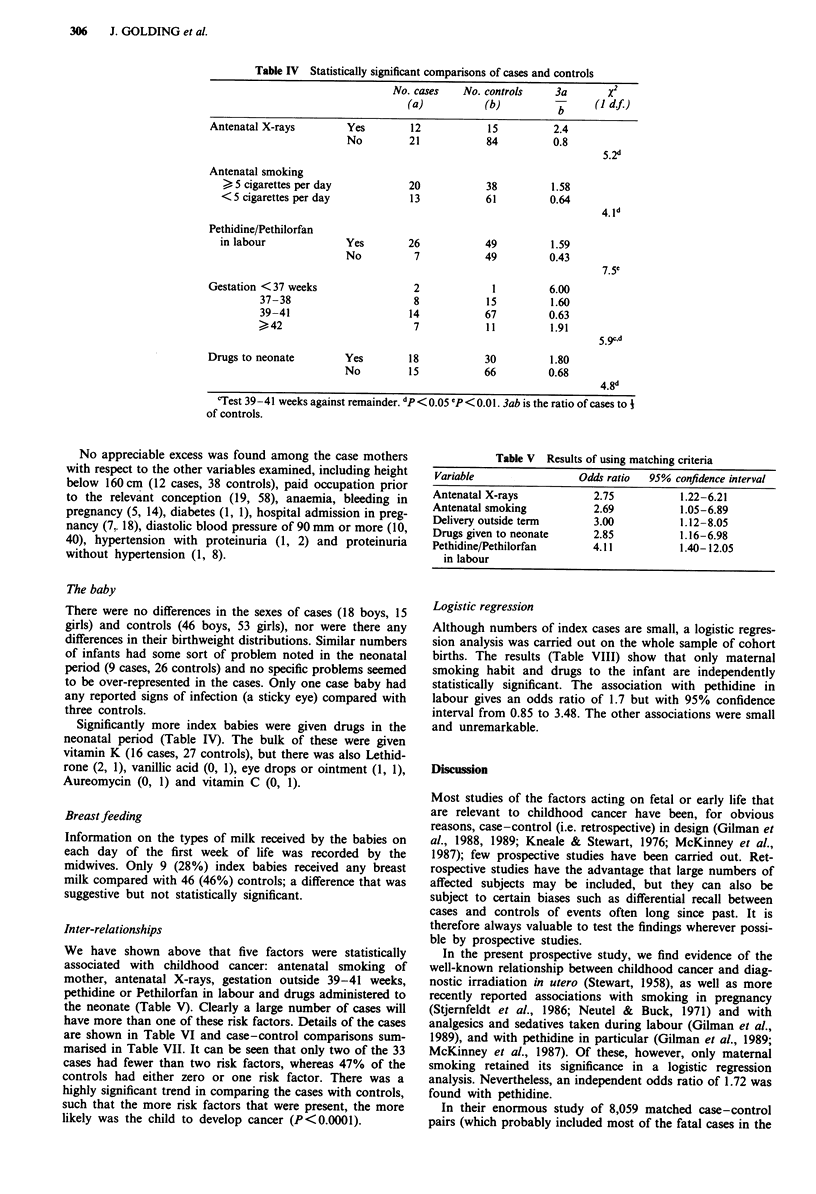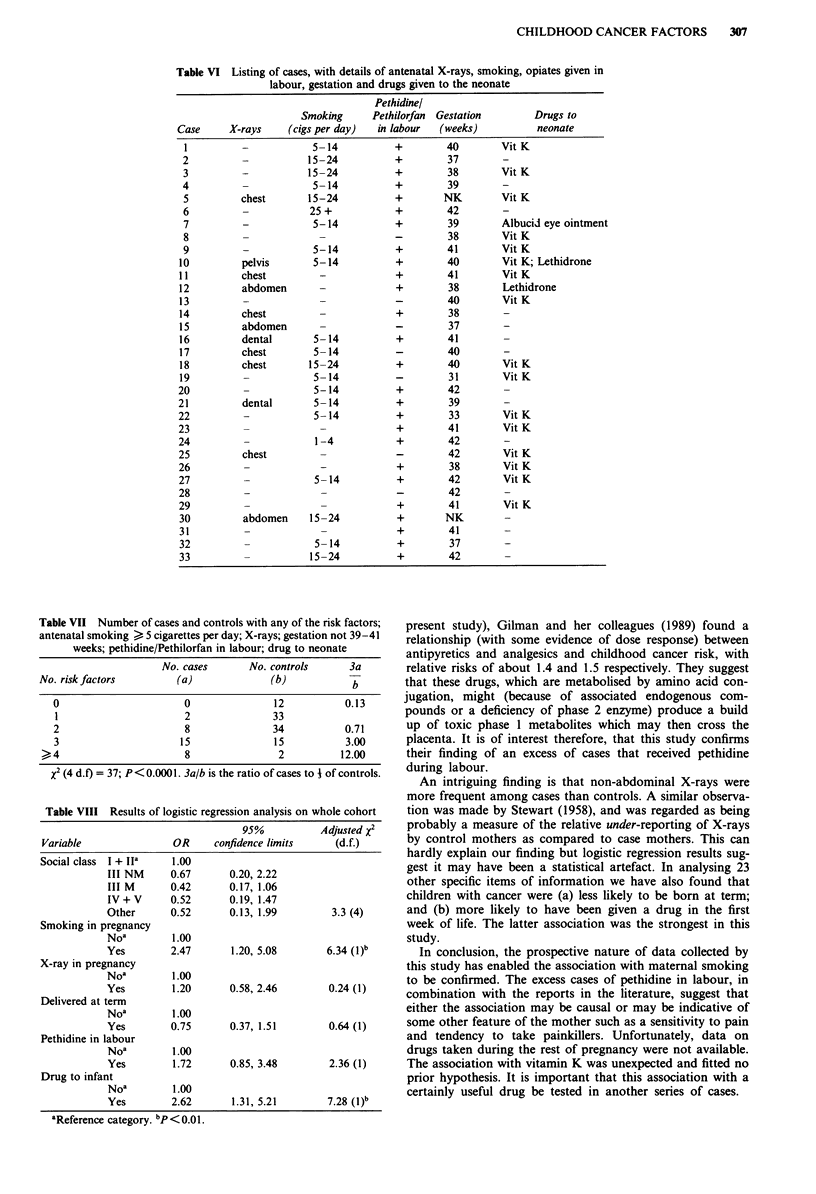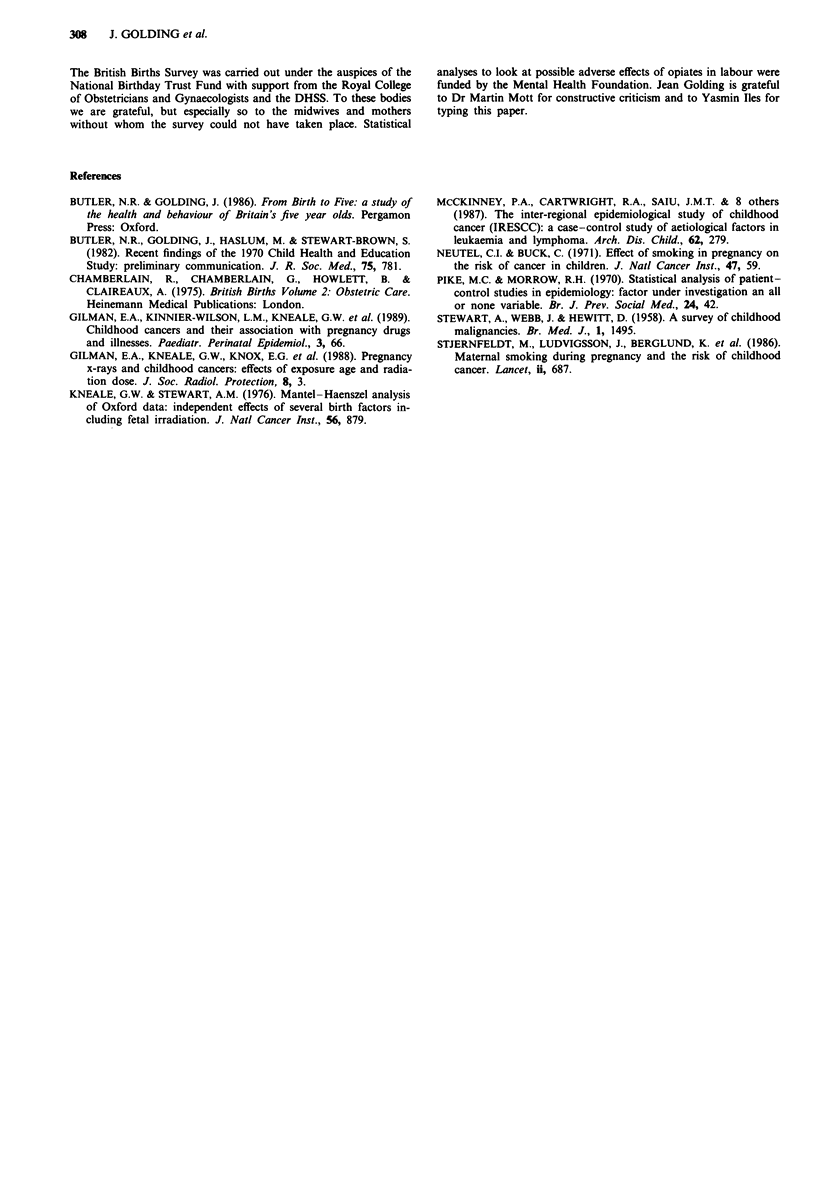# Factors associated with childhood cancer in a national cohort study.

**DOI:** 10.1038/bjc.1990.283

**Published:** 1990-08

**Authors:** J. Golding, M. Paterson, L. J. Kinlen

**Affiliations:** Department of Child Health, Royal Hospital for Sick Children, St Michael's Hill, Bristol, UK.

## Abstract

Information on 16,193 infants delivered in Great Britain in one week of April, 1970 was collected by midwives at the birth and during the first 7 days of life. Using multiple sources, 33 children developing cancer by 1980 were identified from this cohort, giving an incidence of 2.04 per 1,000 total births by the age of 10. Comparisons of these 33 children were made with 99 controls, three for each index case, matched on maternal age, parity and social class. Statistically significant associations were initially found with maternal X-rays and smoking during pregnancy, and the use of analgesics such as pethidine during labour, confirming the findings of retrospective case-control studies. Unexpected statistically significant associations were found with delivery of the child outside term, and drug administration in the first week of life. The latter was found in the absence of an association with neonatal abnormalities in the child and relates mostly to the administration of prophylactic drugs such as vitamin K. Logistic regression involving the whole cohort showed independent statistical associations with maternal smoking (OR 2.5), and drugs to the infant (OR 2.6). After adjusting for these factors no other statistically significant associations were found.


*Br. J. Cancer* (1990), **62**, 304–308 © Macmillan Press Ltd., 1990

### Factors associated with childhood cancer in a national cohort study

J. Golding[1], M. Paterson[1] & L.J. Kinlen[2]

[1]*Department of Child Health, Royal Hospital for Sick Children, St Michael's Hill, Bristol BS2 8BJ, UK; and* [2]*CRC Cancer Epidemiology Unit, The Link Building, 15 George Square, Edinburgh EH8 9JZ, UK.*

**Summary** Information on 16,193 infants delivered in Great Britain in one week of April, 1970 was collected by midwives at the birth and during the first 7 days of life. Using multiple sources, 33 children developing cancer by 1980 were identified from this cohort, giving an incidence of 2.04 per 1,000 total births by the age of 10. Comparisons of these 33 children were made with 99 controls, three for each index case, matched on maternal age, parity and social class. Statistically significant associations were initially found with maternal X-rays and smoking during pregnancy, and the use of analgesics such as pethidine during labour, confirming the findings of retrospective case–control studies. Unexpected statistically significant associations were found with delivery of the child outside term, and drug administration in the first week of life. The latter was found in the absence of an association with neonatal abnormalities in the child and relates mostly to the administration of prophylactic drugs such as vitamin K. Logistic regression involving the whole cohort showed independent statistical associations with maternal smoking (OR 2.5), and drugs to the infant (OR 2.6). After adjusting for these factors no other statistically significant associations were found.

The Oxford Survey of Childhood Cancers (Gilman *et al.*, 1988, 1989) has recently reported retrospective findings on 8,059 children who died of cancer and a similar number of controls. Apart from the well known excess of diagnostic X-rays in the case group an association was found between childhood cancer and pethidine and other analgesics in the relevant pregnancy. Regression analyses suggested that viral infections, vaccines, antipyretics and analgesics were each independently associated with childhood cancer.

Case–control studies may be affected by differences in recall between the study groups, as well as by other forms of bias. Furthermore, population studies have shown that drug consumption during pregnancy is higher than is recalled in case–control studies. We have therefore examined relevant information that was collected prospectively on over 16,000 pregnancies in 1970 in relation to subsequent cancer in childhood.

### Materials and methods

Information on 16,193 infants delivered in Great Britain in one week of April 1970 was collected by midwives at the birth of the child and during the first 7 days of life (Chamberlain *et al.*, 1975). These children were followed up at ages 5 and 10 by the Child Health and Education Study. In all 80% and 94% respectively were successfully contacted (Butler *et al.*, 1982; Butler & Golding, 1986). Deaths among children born in the study week were identified and the relevant death certificates traced.

### *Identification of cases*

Cases of cancer in the cohort were identified in three ways: from death certificates, through the Cancer Registration scheme, and at the follow-up interviews at the ages of 5 and 10. Medical records concerning children treated for any condition which might have been malignant were obtained from the consultants concerned. Two other neoplasms that were ascertained by the above methods were also included, a congenital angioma regarded by the cancer registry as malignant and also a benign dermoid teratoma.

### *Control sample*

Computerised matching procedures were used to produce a set of three controls for each case, matched for the following factors: age of the mother at the birth of the child, parity and social class (based on the occupation of the mother's husband at the time of the birth), marital status at delivery, and whether the birth was single or multiple.

### *Statistical methods*

Case–control analysis has used the matching to test for statistical significance, but for ease of interpretation the results presented compare the proportions within each group. For computation of odds ratios and their 95% confidence intervals the full matched quadruplets have been used with the method described by Pike and Morrow (1970).

### Results

In all there were 33 children who developed cancer by the age of 10 out of a total population of 16,193 births in Great Britain occurring in the survey week of 1970: an incidence of 2.04 per 1,000 total births. Details of ascertainment, diagnosis and outcome are listed in Table I. As already noted, there were two cases which may not, strictly speaking have been cancer. Omitting these would result in an incidence of 1.91.

The maternal age, parity and social class distributions are compared with the total population of births in Table II. There was a social class gradient with a higher incidence in social classes I and II (the professional and managerial) than in IV and V (the semi-skilled and unskilled workers) but this was not statistically significant. No relationship was noted with either parity or maternal age. A satisfactory matching of controls was achieved (Table III).

### *X-rays*

Information on X-ray exposure in pregnancy is shown in Table IV. There was a statistically significant excess of index mothers having had X-rays, but the results were similar for X-rays involving the abdomen (4 cases, 5 controls) as for chest or dental X-rays (8 cases, 10 controls).

### *Labour and delivery*

Similar proportions had labour induced (10 cases, 22 controls), had first stages in excess of 15 hours (5 cases, 15 controls) or second stages in excess of 60 minutes (3 cases, 10 controls). Caesarean section was carried out for one case and five controls.

Despite similar durations of labour among cases and controls, there was a difference in the numbers given analgesics or

Correspondence: J. Golding.
Received 3 August 1989; and in revised form 22 February 1990.

**Table I** Cases of childhood cancer identified by the time the child was 10

| *Reference number* | *Cancer* | *Age (yr) at diagnosis* | *Outcome* | *Sources of ascertainment* |
|---|---|---|---|---|
| *Leukaemia* | | | | |
| 00463 | Acute lymphoblastic | NK | Died aged 9 yrs | C.V. |
| 02005 | Acute lymphoblastic | 4 | Died aged 6 yrs | C.V. |
| 02258 | Chronic myeloid | 1 | Died at 18 mths | D.C. |
| 02334 | Acute lymphoblastic | 6 | Alive at 10 | C.V. |
| 06331 | Not stated | 0 | Died aged 5 mths | D.V. |
| 08447 | Acute lymphoblastic | 5 | Alive at 7 | C.V. |
| 10553 | Lymphoblastic | 10 | Died aged 10 yrs | V |
| 12489 | Acute lymphoblastic | 8 | Alive at 10 | C |
| 14116 | Acute lymphatic | 3 | Died aged 3 yrs | D.V. |
| *Lymphoma* | | | | |
| 00304 | Non-Hodgkin's | 9 | Alive at 10 | C |
| 04150 | Non-Hodgkin's | 8 | Died aged 8 yrs | D.V. |
| 13162 | Non-Hodgkin's | 9 | Alive at 10 | C.V. |
| 14165 | Lymphocytic | 5 | Alive at 10 | C |
| 01196 | Haemangioendotheliosis | 0 | Died aged 3 wks | D.V. |
| *Brain* | | | | |
| 05740 | Medulloblastoma | 8 | Died aged 8 yrs | D.V. |
| 12767 | Brain stem tumour | 5 | Died aged 5 yrs | D.V. |
| 12973 | Cerebral tumour | 0 | Died aged 9 mths | D.V. |
| 13208 | Congenital angioma | 2 | Died aged 2 yrs | D.V. |
| 14499 | Medulloblastoma | 5 | Died aged 5 yrs | D.V. |
| 14876 | Cerebellar tumour | 2 | Died aged 2 yrs | D.V. |
| 08412(1) | Astrocytoma | 7 | Died aged 8 yrs | D.V. |
| 15400 | Astrocytoma | 9 | Alive at 10 | C |
| *Eye* | | | | |
| 08916 | Bilateral retinoblastoma | 0 | Alive at 10 | C.V. |
| 09106 | Retinoblastoma | 1 | Alive at 10 | C.V. |
| 13248 | Retinoblastoma | 0 | Alive at 10 | C.V. |
| *Bone* | | | | |
| 16188 | Sarcoma of humerus | 10 | Died aged 11 yrs | D |
| *Kidney* | | | | |
| 02997 | Wilm's tumour | 4 | Alive at 10 | C.V. |
| 05947 | Wilm's tumour | 2 | Alive at 10 | C.V. |
| 06313 | Wilm's tumour | 3 | Died aged 3 yrs | D.V. |
| 14416 | Wilm's tumour | 3 | Alive at 10 | C.V. |
| 14907 | Wilm's tumour | 3 | Alive at 10 | C.V. |
| *Teratoma* | | | | |
| 05852 | Benign dermoid | 4 | Alive at 10 | C |
| 11999 | Sacrococcygeal | 3 | Died aged 3 yrs | D.V. |

V, validated from various sources. D, Death certificates obtained by Child Health & Education Study. C, Maternal interview and hospital records obtained by Child Health & Education Study.

**Table II** Incidence of cancer (per 1,000 total births) by maternal age, parity and social class

| *Maternal age* | | *Parity* | | *Social class* | |
|---|---|---|---|---|---|
| < 20 | 2.5 (4) | 0 | 1.6 (10) | I | 6.5 (5) |
| 20–24 | 1.9 (10) | 1 | 2.1 (10) | II | 2.2 (4) |
| 25–29 | 2.2 (11) | 2 | 2.7 (7) | III | 1.7 (14) |
| 30–34 | 2.5 (6) | 3 | 1.7 (2) | IV | 1.7 (4) |
| ≥35 + | 0.8 (1) | ≥4 | 2.8 (3) | V | 1.1 (2) |
| | | | | Other | 5.7 (3) |
| All | 2.0 (33) | | 2.0 (33) | | 2.0 (33) |

Numbers with cancer in parentheses.

**Table III** Distribution of cases and controls on matching criteria

| *Maternal age* | *CS* | *CT* | *Parity* | *CS* | *CT* | *Social class* | *CS* | *CT* |
|---|---|---|---|---|---|---|---|---|
| <20 | 4 | 12 | 0 | 10 | 30 | I | 5 | 15 |
| 20–24 | 11 | 33 | 1 | 11 | 33 | II | 4 | 12 |
| 25–29 | 11 | 34 | 2 | 7 | 21 | III | 15 | 45 |
| 30–34 | 6 | 17 | 3 | 2 | 6 | IV | 4 | 12 |
| ≥35 | 1 | 3 | 4+ | 3 | 9 | V | 2 | 6 |
| | | | | | | Other | 3 | 9 |
| Total | 33 | 99 | | 33 | 99 | | 33 | 99 |

CS, index case. CT, matched controls.

sedatives in labour (27 cases and 59 controls, $P<0.05$). Only two specific drugs in this group were given in sufficient numbers to allow statistical comparison (Table IV): pethidine (19 cases, 29 controls, $P<0.01$) and Pethilorfan, a pethidine containing drug (7 cases, 21 controls). In all, 26 index mothers were given a pethidine preparation in labour compared with 49 controls (one control mother had been given both pethidine and Pethilorfan), a significant difference ($P<0.01$).

Details of the length of gestation are shown in Table IV. There were significantly fewer index mothers delivered at term (39–41 weeks), with excess numbers seen in the weeks both before and after this period.

### *Other maternal aspects*

Compared to the controls, more mothers of cases had smoked 5 cigarettes or more per day throughout pregnancy (20 cases, 38 controls, $P<0.05$). Slightly fewer case than control mothers had reported using the contraceptive pill in the 18 months prior to conception (8 cases, 29 controls). A significant deficiency of blood group A was noted in the case mothers (8/31 cases, 45/91 controls; $\chi^2=4.5$, $P<0.05$), but did not persist in the matched analysis. The numbers of mothers who were Rhesus negative were similar (6 cases, 17 controls).

**Table IV** Statistically significant comparisons of cases and controls

| | | *No. cases (a)* | *No. controls (b)* | $\frac{3a}{b}$ | $\chi^2$ *(1 d.f.)* |
|---|---|---|---|---|---|
| Antenatal X-rays | Yes | 12 | 15 | 2.4 | |
| | No | 21 | 84 | 0.8 | |
| | | | | | 5.2[d] |
| Antenatal smoking | | | | | |
| ⩾5 cigarettes per day | | 20 | 38 | 1.58 | |
| <5 cigarettes per day | | 13 | 61 | 0.64 | |
| | | | | | 4.1[d] |
| Pethidine/Pethilorfan in labour | Yes | 26 | 49 | 1.59 | |
| | No | 7 | 49 | 0.43 | |
| | | | | | 7.5[e] |
| Gestation <37 weeks | | 2 | 1 | 6.00 | |
| 37–38 | | 8 | 15 | 1.60 | |
| 39–41 | | 14 | 67 | 0.63 | |
| ⩾42 | | 7 | 11 | 1.91 | |
| | | | | | 5.9[c,d] |
| Drugs to neonate | Yes | 18 | 30 | 1.80 | |
| | No | 15 | 66 | 0.68 | |
| | | | | | 4.8[d] |

[c]Test 39–41 weeks against remainder. [d]$P<0.05$ [e]$P<0.01$. *3ab* is the ratio of cases to ⅓ of controls.

No appreciable excess was found among the case mothers with respect to the other variables examined, including height below 160 cm (12 cases, 38 controls), paid occupation prior to the relevant conception (19, 58), anaemia, bleeding in pregnancy (5, 14), diabetes (1, 1), hospital admission in pregnancy (7, 18), diastolic blood pressure of 90 mm or more (10, 40), hypertension with proteinuria (1, 2) and proteinuria without hypertension (1, 8).

### *The baby*

There were no differences in the sexes of cases (18 boys, 15 girls) and controls (46 boys, 53 girls), nor were there any differences in their birthweight distributions. Similar numbers of infants had some sort of problem noted in the neonatal period (9 cases, 26 controls) and no specific problems seemed to be over-represented in the cases. Only one case baby had any reported signs of infection (a sticky eye) compared with three controls.

Significantly more index babies were given drugs in the neonatal period (Table IV). The bulk of these were given vitamin K (16 cases, 27 controls), but there was also Lethidrone (2, 1), vanillic acid (0, 1), eye drops or ointment (1, 1), Aureomycin (0, 1) and vitamin C (0, 1).

### *Breast feeding*

Information on the types of milk received by the babies on each day of the first week of life was recorded by the midwives. Only 9 (28%) index babies received any breast milk compared with 46 (46%) controls; a difference that was suggestive but not statistically significant.

### *Inter-relationships*

We have shown above that five factors were statistically associated with childhood cancer: antenatal smoking of mother, antenatal X-rays, gestation outside 39–41 weeks, pethidine or Pethilorfan in labour and drugs administered to the neonate (Table V). Clearly a large number of cases will have more than one of these risk factors. Details of the cases are shown in Table VI and case–control comparisons summarised in Table VII. It can be seen that only two of the 33 cases had fewer than two risk factors, whereas 47% of the controls had either zero or one risk factor. There was a highly significant trend in comparing the cases with controls, such that the more risk factors that were present, the more likely was the child to develop cancer ($P<0.0001$).

**Table V** Results of using matching criteria

| *Variable* | *Odds ratio* | *95% confidence interval* |
|---|---|---|
| Antenatal X-rays | 2.75 | 1.22–6.21 |
| Antenatal smoking | 2.69 | 1.05–6.89 |
| Delivery outside term | 3.00 | 1.12–8.05 |
| Drugs given to neonate | 2.85 | 1.16–6.98 |
| Pethidine/Pethilorfan in labour | 4.11 | 1.40–12.05 |

### *Logistic regression*

Although numbers of index cases are small, a logistic regression analysis was carried out on the whole sample of cohort births. The results (Table VIII) show that only maternal smoking habit and drugs to the infant are independently statistically significant. The association with pethidine in labour gives an odds ratio of 1.7 but with 95% confidence interval from 0.85 to 3.48. The other associations were small and unremarkable.

### Discussion

Most studies of the factors acting on fetal or early life that are relevant to childhood cancer have been, for obvious reasons, case–control (i.e. retrospective) in design (Gilman *et al.*, 1988, 1989; Kneale & Stewart, 1976; McKinney *et al.*, 1987); few prospective studies have been carried out. Retrospective studies have the advantage that large numbers of affected subjects may be included, but they can also be subject to certain biases such as differential recall between cases and controls of events often long since past. It is therefore always valuable to test the findings wherever possible by prospective studies.

In the present prospective study, we find evidence of the well-known relationship between childhood cancer and diagnostic irradiation *in utero* (Stewart, 1958), as well as more recently reported associations with smoking in pregnancy (Stjernfeldt *et al.*, 1986; Neutel & Buck, 1971) and with analgesics and sedatives taken during labour (Gilman *et al.*, 1989), and with pethidine in particular (Gilman *et al.*, 1989; McKinney *et al.*, 1987). Of these, however, only maternal smoking retained its significance in a logistic regression analysis. Nevertheless, an independent odds ratio of 1.72 was found with pethidine.

In their enormous study of 8,059 matched case–control pairs (which probably included most of the fatal cases in the

**Table VI** Listing of cases, with details of antenatal X-rays, smoking, opiates given in labour, gestation and drugs given to the neonate

| *Case* | *X-rays* | *Smoking (cigs per day)* | *Pethidine/ Pethilorfan in labour* | *Gestation (weeks)* | *Drugs to neonate* |
|---|---|---|---|---|---|
| 1 | – | 5–14 | + | 40 | Vit K |
| 2 | – | 15–24 | + | 37 | – |
| 3 | – | 15–24 | + | 38 | Vit K |
| 4 | – | 5–14 | + | 39 | – |
| 5 | chest | 15–24 | + | NK | Vit K |
| 6 | – | 25+ | + | 42 | – |
| 7 | – | 5–14 | + | 39 | Albucid eye ointment |
| 8 | – | – | – | 38 | Vit K |
| 9 | – | 5–14 | + | 41 | Vit K |
| 10 | pelvis | 5–14 | + | 40 | Vit K; Lethidrone |
| 11 | chest | – | + | 41 | Vit K |
| 12 | abdomen | – | + | 38 | Lethidrone |
| 13 | – | – | – | 40 | Vit K |
| 14 | chest | – | + | 38 | – |
| 15 | abdomen | – | – | 37 | – |
| 16 | dental | 5–14 | + | 41 | – |
| 17 | chest | 5–14 | – | 40 | – |
| 18 | chest | 15–24 | + | 40 | Vit K |
| 19 | – | 5–14 | – | 31 | Vit K |
| 20 | – | 5–14 | + | 42 | – |
| 21 | dental | 5–14 | + | 39 | – |
| 22 | – | 5–14 | + | 33 | Vit K |
| 23 | – | – | + | 41 | Vit K |
| 24 | – | 1–4 | + | 42 | – |
| 25 | chest | – | – | 42 | Vit K |
| 26 | – | – | + | 38 | Vit K |
| 27 | – | 5–14 | + | 42 | Vit K |
| 28 | – | – | – | 42 | – |
| 29 | – | – | + | 41 | Vit K |
| 30 | abdomen | 15–24 | + | NK | – |
| 31 | – | – | + | 41 | – |
| 32 | – | 5–14 | + | 37 | – |
| 33 | – | 15–24 | + | 42 | – |

**Table VII** Number of cases and controls with any of the risk factors; antenatal smoking $\geqslant 5$ cigarettes per day; X-rays; gestation not 39–41 weeks; pethidine/Pethilorfan in labour; drug to neonate

| *No. risk factors* | *No. cases (a)* | *No. controls (b)* | $\frac{3a}{b}$ |
|---|---|---|---|
| 0 | 0 | 12 | 0.13 |
| 1 | 2 | 33 | |
| 2 | 8 | 34 | 0.71 |
| 3 | 15 | 15 | 3.00 |
| $\geqslant 4$ | 8 | 2 | 12.00 |

$\chi^2$ (4 d.f) = 37; $P<0.0001$. *3a/b* is the ratio of cases to ⅓ of controls.

**Table VIII** Results of logistic regression analysis on whole cohort

| *Variable* | | *OR* | *95% confidence limits* | *Adjusted $\chi^2$* (d.f.) |
|---|---|---|---|---|
| Social class | I + II[a] | 1.00 | | |
| | III NM | 0.67 | 0.20, 2.22 | |
| | III M | 0.42 | 0.17, 1.06 | |
| | IV + V | 0.52 | 0.19, 1.47 | |
| | Other | 0.52 | 0.13, 1.99 | 3.3 (4) |
| Smoking in pregnancy | | | | |
| | No[a] | 1.00 | | |
| | Yes | 2.47 | 1.20, 5.08 | 6.34 (1)[b] |
| X-ray in pregnancy | | | | |
| | No[a] | 1.00 | | |
| | Yes | 1.20 | 0.58, 2.46 | 0.24 (1) |
| Delivered at term | | | | |
| | No[a] | 1.00 | | |
| | Yes | 0.75 | 0.37, 1.51 | 0.64 (1) |
| Pethidine in labour | | | | |
| | No[a] | 1.00 | | |
| | Yes | 1.72 | 0.85, 3.48 | 2.36 (1) |
| Drug to infant | | | | |
| | No[a] | 1.00 | | |
| | Yes | 2.62 | 1.31, 5.21 | 7.28 (1)[b] |

[a]Reference category. [b]$P<0.01$.

present study), Gilman and her colleagues (1989) found a relationship (with some evidence of dose response) between antipyretics and analgesics and childhood cancer risk, with relative risks of about 1.4 and 1.5 respectively. They suggest that these drugs, which are metabolised by amino acid conjugation, might (because of associated endogenous compounds or a deficiency of phase 2 enzyme) produce a build up of toxic phase 1 metabolites which may then cross the placenta. It is of interest therefore, that this study confirms their finding of an excess of cases that received pethidine during labour.

An intriguing finding is that non-abdominal X-rays were more frequent among cases than controls. A similar observation was made by Stewart (1958), and was regarded as being probably a measure of the relative *under*-reporting of X-rays by control mothers as compared to case mothers. This can hardly explain our finding but logistic regression results suggest it may have been a statistical artefact. In analysing 23 other specific items of information we have also found that children with cancer were (a) less likely to be born at term; and (b) more likely to have been given a drug in the first week of life. The latter association was the strongest in this study.

In conclusion, the prospective nature of data collected by this study has enabled the association with maternal smoking to be confirmed. The excess cases of pethidine in labour, in combination with the reports in the literature, suggest that either the association may be causal or may be indicative of some other feature of the mother such as a sensitivity to pain and tendency to take painkillers. Unfortunately, data on drugs taken during the rest of pregnancy were not available. The association with vitamin K was unexpected and fitted no prior hypothesis. It is important that this association with a certainly useful drug be tested in another series of cases.

The British Births Survey was carried out under the auspices of the National Birthday Trust Fund with support from the Royal College of Obstetricians and Gynaecologists and the DHSS. To these bodies we are grateful, but especially so to the midwives and mothers without whom the survey could not have taken place. Statistical analyses to look at possible adverse effects of opiates in labour were funded by the Mental Health Foundation. Jean Golding is grateful to Dr Martin Mott for constructive criticism and to Yasmin Iles for typing this paper.

### References

BUTLER, N.R. & GOLDING, J. (1986). *From Birth to Five: a study of the health and behaviour of Britain's five year olds*. Pergamon Press: Oxford.

BUTLER, N.R., GOLDING, J., HASLUM, M. & STEWART-BROWN, S. (1982). Recent findings of the 1970 Child Health and Education Study: preliminary communication. *J. R. Soc. Med.*, **75**, 781.

CHAMBERLAIN, R., CHAMBERLAIN, G., HOWLETT, B. & CLAIREAUX, A. (1975). *British Births Volume 2: Obstetric Care*. Heinemann Medical Publications: London.

GILMAN, E.A., KINNIER-WILSON, L.M., KNEALE, G.W. *et al.* (1989). Childhood cancers and their association with pregnancy drugs and illnesses. *Paediatr. Perinatal Epidemiol.*, **3**, 66.

GILMAN, E.A., KNEALE, G.W., KNOX, E.G. *et al.* (1988). Pregnancy x-rays and childhood cancers: effects of exposure age and radiation dose. *J. Soc. Radiol. Protection*, **8**, 3.

KNEALE, G.W. & STEWART, A.M. (1976). Mantel–Haenszel analysis of Oxford data: independent effects of several birth factors including fetal irradiation. *J. Natl Cancer Inst.*, **56**, 879.

MCCKINNEY, P.A., CARTWRIGHT, R.A., SAIU, J.M.T. & 8 others (1987). The inter-regional epidemiological study of childhood cancer (IRESCC): a case–control study of aetiological factors in leukaemia and lymphoma. *Arch. Dis. Child.*, **62**, 279.

NEUTEL, C.I. & BUCK, C. (1971). Effect of smoking in pregnancy on the risk of cancer in children. *J. Natl Cancer Inst.*, **47**, 59.

PIKE, M.C. & MORROW, R.H. (1970). Statistical analysis of patient–control studies in epidemiology: factor under investigation an all or none variable. *Br. J. Prev. Social Med.*, **24**, 42.

STEWART, A., WEBB, J. & HEWITT, D. (1958). A survey of childhood malignancies. *Br. Med. J.*, **1**, 1495.

STJERNFELDT, M., LUDVIGSSON, J., BERGLUND, K. *et al.* (1986). Maternal smoking during pregnancy and the risk of childhood cancer. *Lancet*, **ii**, 687.